# Preference versus protocol: oncology clinicians’ perspectives on central venous access for administration of chemotherapy in pancreatic cancer

**DOI:** 10.1016/j.esmogo.2026.100311

**Published:** 2026-03-02

**Authors:** M.U.J.E. Graus, R.A.L. Willems, N.C. Biesma, A.J. de Wilde, F.W.P.J. van den Berkmortel, S.A.W. Bouwense, G.A. Cirkel, M.Y.V. Homs, E. Jellema-Betten, N. Pepels-Aarts, H.C. van Santvoort, E.C.J. van Vliet, M.L. Wumkes, J.W. Wilmink, I.H.J.T. de Hingh, L.B.J. Valkenburg-van Iersel, J. de Vos-Geelen

**Affiliations:** 1 Division of Medical Oncology, Department of Internal Medicine, Maastricht University Medical Center, Maastricht, The Netherlands; 2 GROW – Research Institute for Oncology & Reproduction, Maastricht University, Maastricht, The Netherlands; 3 Department of Surgery, University Medical Center Utrecht Cancer Center & St. Antonius Hospital Nieuwegein, Regional Academic Cancer Center Utrecht, Utrecht, The Netherlands; 4 Department of Surgery, School of Nutrition and Translational Research in Metabolism (NUTRIM), Maastricht University Medical Center, Maastricht University, Maastricht, The Netherlands; 5 Department of Medical Oncology, Zuyderland Medical Centre, Sittard-Geleen, The Netherlands; 6 Department of Medical Oncology, Utrecht, University Medical Center Utrecht Cancer Center & St. Antonius Hospital Nieuwegein, Regional Academic Cancer Center Utrecht, The Netherlands; 7 Department of Medical Oncology, Erasmus MC Cancer Institute, Rotterdam, The Netherlands; 8 Division of Hepatobiliary Surgery, Department of Surgery, University Medical Center Groningen, Groningen, The Netherlands; 9 Division of Medical Oncology, Department of Internal Medicine, Admiraal de Ruyter Hospital (ADRZ), Vlissingen, The Netherlands; 10 Division of Medical Oncology, Department of Internal Medicine, Jeroen Bosch Hospital, Den Bosch, The Netherlands; 11 Department of Medical Oncology, Amsterdam University Medical Center, University of Amsterdam, Amsterdam, The Netherlands; 12 Department of Surgery, Catharina Hospital Eindhoven, Eindhoven, The Netherlands

**Keywords:** pancreas adenocarcinoma, quality of life, 5-fluorouracil, port-a-cath, peripheral inserted central catheter

## Abstract

**Background:**

Pancreatic cancer treatment significantly impacts patients’ quality of life, making both safety and patient preference key considerations. Central venous access devices (CVADs) are indispensable for chemotherapy administration in pancreatic cancer, yet device selection varies widely. This study explored which CVADs oncology specialists use in pancreatic cancer care, focusing on the basis for their recommendations.

**Materials and methods:**

A nationwide expert survey was distributed among Dutch medical oncologists and nurse specialists involved in pancreatic cancer care via the Dutch Pancreatic Cancer Group, the Dutch Association for Medical Oncology, the Dutch association for nurses, and the study committee’s network.

**Results:**

Ninety-one clinicians responded. Most (88%) had access to both port-a-caths (PORTs) and peripherally inserted central catheters (PICCs), while 12% could only offer one device. Decision-making autonomy varied: 53% reported full autonomy, while others followed hospital-wide preferences (39%) or guidelines (9%). Even within these subgroups, preferred CVAD varied greatly. Although 60% listed patient preference among the top five influential factors, only 28% incorporated patients in that decision. Logistical constraints were key barriers influencing device choice.

**Conclusion:**

Substantial variability exists in CVAD selection, availability, and clinician autonomy in pancreatic cancer care. While evidence supports PORTs as the safer option, PICCs remain widely used in daily practice. This discrepancy appears driven by disease-specific and logistical factors, including poor prognosis and uncertainty regarding treatment tolerance. Addressing real-world barriers through improved access to PORTs, clearer guideline recommendations, and enhanced patient counseling may help align clinical practice with evidence and ensure high-quality care for patients receiving chemotherapy.

## Highlights

• Nearly half primarily recommend PICCs for pancreatic cancer chemotherapy.
• Logistical delays and sedation needs are key barriers to PORT use.
• 60% rated patient preference among top factors, but only 28% involve patients in choice.
• Wide variability exists in CVAD selection, availability and clinician autonomy.

## Introduction

Pancreatic cancer is an aggressive form of cancer with one of the highest cancer-related mortality rates worldwide.^1^^,^^2^ More than half of patients present with advanced disease at diagnosis, leaving them with solely palliative, life-prolonging options.^3^ Given the substantial impact these limited, but intensive, treatment options can have on quality of life, attention has increasingly shifted not only to therapeutic efficacy, but also the safety and feasibility of treatment administration.

Fluorouracil, leucovorin, irinotecan, and oxaliplatin (FOLFIRINOX) is a universally accepted first-line chemotherapy regimen in both (neo)adjuvant and palliative settings for eligible patients.^4^, ^5^, ^6^, ^7^, ^8^, ^9^ However, this regimen also involves significant toxicity and a time-consuming administration schedule, including a 46-h continuous infusion of 5-fluorouracil (5-FU). This prioritizes the use of a central venous access device (CVAD) to allow home-based treatment. The same applies to the accepted second-line therapy after progression on gemcitabine/nab-paclitaxel, nanoliposomal irinotecan plus 5-FU.

The most commonly used CVADs are fully implantable catheters (PORTs, port-a-caths, or PACs) and peripherally inserted central catheters (PICCs).^10^ Particularly in the palliative setting, CVADs should provide both safe and easy access, low morbidity, and high patient acceptance rates. Evidence demonstrates PORTs are associated with lower complication rates for both venous thrombosis and infections, as well as less frequent malfunctioning issues.^11^, ^12^, ^13^, ^14^, ^15^, ^16^ While evidence regarding cost differences is mixed, PORTs are generally deemed cost-effective given the fewer complications.^11^^,^^12^^,^^16^^,^^17^ However, PORT placement is more invasive, often requiring sedation or anesthesia, and typically involves longer waiting times.

Despite these findings, PICCs are still regularly used and there is no consensus or standardized guideline for CVAD use in cancer care.^18^ It is currently unclear what other factors, apart from complication risk, clinicians base their preferences and recommendations on and whether shared decision-making (SDM) is applied. This study aimed to investigate which CVADs oncology specialists use for administering chemotherapy in pancreatic cancer patients, the rationale behind their recommendations, and the extent to which patient preference plays a role in clinical decision making.

## Materials and methods

### Study design and database

A nationwide expert survey was conducted using a 31-item questionnaire developed by the study protocol committee. The survey was anonymous and hosted on Qualtrics, with the final version (26-08-2024) available in the Supplementary Material (Supplementary Table S1, available at https://doi.org/10.1016/j.esmogo.2026.100311). Approval was obtained from the Scientific Committee of the Dutch Pancreatic Cancer Group (DPCG).^19^

Medical oncologists, nurse specialists, and physician assistants involved in pancreatic cancer care were invited to participate as they are primarily responsible for discussing CVAD options with patients and initiating referrals for device placement. Clinicians were invited via the DPCG, the Dutch Association for Medical Oncology (NVMO), the Dutch association for nurses, caregivers, and nurse specialists (V&VN), and the study committee’s network. Participants were contacted via email and encouraged to share the survey with fellow medical oncologists and/or nurse specialists. Two targeted reminders were sent.

### Study definitions

Participants could select among the following CVADs: PORTs, PICCs, Hickman catheters (HCs), or ‘other’, and had the opportunity to elaborate upon their answers. Based on their reported prescribing patterns, respondents were categorized as having a preferred PORT or PICC practice (>75% use), or as having a somewhat equal preference if both devices were recommended in >25% but neither exceeded 75%. This categorization allowed comparison of decision-making factors and institutional practices.

### Statistical analysis

Descriptive statistics were employed to characterize the demographic attributes of respondents. Categorical data are presented as numbers and percentages and continuous data as means with standard deviation (SD) or medians with interquartile range (IQR). Subgroup comparisons were conducted using chi-square or Fisher’s exact tests. Analyses were carried out using IBM SPSS Statistics for Windows, version 28.0.0.0 (IBM Corp., Armonk, NY), with *P* values <0.05 considered statistically significant.

## Results

### Demographics of respondents

In total, 126 responses were collected between 1 November 2024 and 28 February 2025. Of the collected surveys, 15 were incomplete (8 excluded due to <50% completion), and 27 were excluded as respondents did not advise on CVADs. Consequently, 91 respondents were included in the analysis.

The majority of respondents were medical oncologists (65%), followed by nurse specialists (23%), and medical oncologists in training (8%, Table 1). Most participants practiced in the Netherlands (97%), while a minority (3%) worked in the Caribbean. Regarding the work environment, the largest group (60%) was employed in nonacademic hospitals, 32% worked in academic hospitals, and 8% in nonacademic tertiary referral centers.

**Table 1:** Demographic characteristics of respondents

	*n* (%)
Profession	91
Medical oncologist	59 (65)
Medical oncologist in training	7 (8)
Nurse specialist	21 (23)
Other^a^	4 (4)
Current working environment	91
Academic hospital	29 (32)
Nonacademic tertiary referral center	7 (8)
Nonacademic hospital	55 (60)
Country of practice	91
The Netherlands	88 (97)
Caribbean	3 (3)
Experience in years	90
<5 years	37 (41)
5-10 years	20 (22)
>10 years	33 (36)
New patients with pancreatic cancer on annual basis	91
<10 patients	41 (45)
10-30 patients	42 (46)
>30 patients	8 (9)

a Other professions included nurse specialists in training (*n* = 1), oncology nurses (*n* = 2) and oncology case managers (*n* = 1).

Respondents had varying levels of experience in treating pancreatic cancer: 41% had <5 years of professional experience, 36% of respondents had >10 years, and 22% had between 5 and 10 years of experience. In terms of patient caseload, 46% of respondents treated between 10 and 30 new patients with pancreatic cancer annually, 45% managed <10 new patients, and 9% >30 new cases per year.

### Availability of CVADs

**Figure 1:**
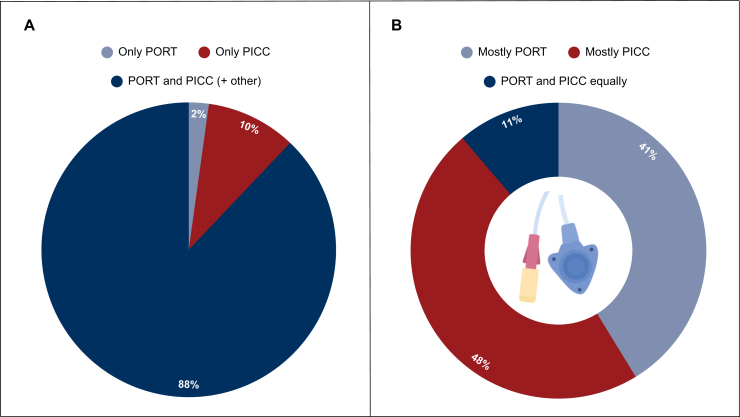
**Available CVAD options and preferences.** (A) CVAD options available at the institution of the health care professional. (B) The CVAD most often selected by health care professionals with multiple CVAD options. CVAD, central venous access device; PICC, peripherally inserted central catheter; PORT, port-a-cath.

Among the 91 respondents, 88% reported having access to both PORTs and PICCs for chemotherapy administration in patients with pancreatic cancer (Figure 1A). Of these, 12% also had access to another CVAD including HCs and arm ports. Eleven professionals (12%) had access to only one type of CVAD, with 2 having access to PORTs exclusively and 10 to PICCs. Of respondents with access to multiple CVADs, 41% most often used PORTs, 48% most often PICCs, and 11% used PORTs and PICCs somewhat equally (Figure 1B). Respondents with access to HCs or other CVADs reported rarely (<25%) or not using these devices.

Decision making regarding CVAD selection varied among respondents. While 53% had full autonomy in choosing a CVAD, others had to follow hospital guidelines (9%) or adhere to a hospital-wide preference with some flexibility (39%, Supplementary Figure S1A, available at https://doi.org/10.1016/j.esmogo.2026.100311). Among those with free choice, 52% most frequently recommended a PICC, while 35% preferred a PORT, and 13% advised both options somewhat equally (Supplementary Figure S1B, available at https://doi.org/10.1016/j.esmogo.2026.100311). Among those who followed hospital guidelines, 51% primarily recommended a PICC, 42% a PORT, and 7% provided both options equally. Respondents adhering to strict hospital guidelines indicated that they would not change their recommendation, even if given full autonomy.

### Factors of influence on CVAD recommendation

**Figure 2:**
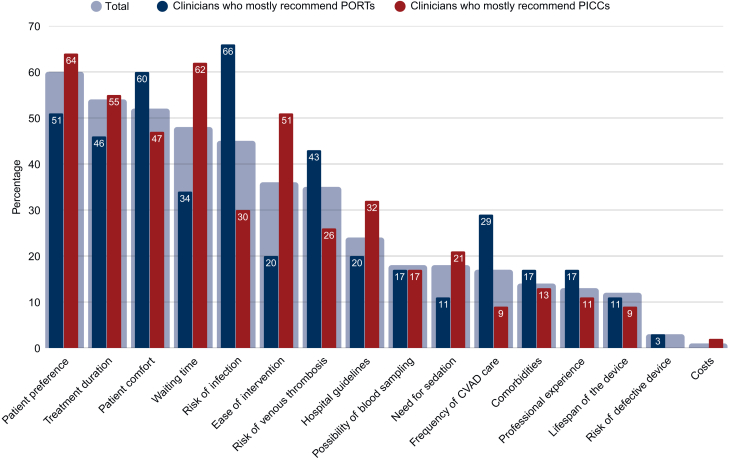
**Factors influencing CVAD selection, stratified by clinicians who primarily recommend PORTs versus PICCs.** The bars represent the proportion of clinicians selecting each factor among their top five considerations. CVAD, central venous access device; PICC, peripherally inserted central catheter; PORT, port-a-cath.

For all respondents, the most frequently cited factor influencing CVAD recommendation was patient preference (60%, Figure 2). Other key considerations in the top five included treatment duration (54%), patient comfort (52%), waiting time for placement (48%), and infection risk (45%).

**Figure 3:**
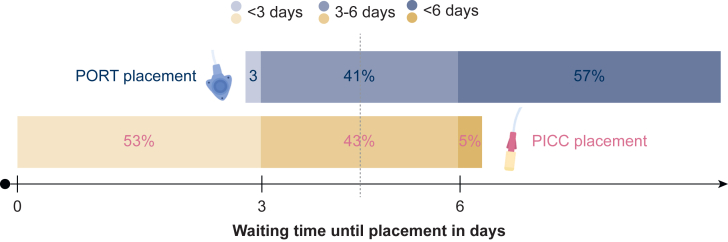
**Waiting time until CVAD placement in days.** CVAD, central venous access device; PICC, peripherally inserted central catheter; PORT, port-a-cath.

Logistical aspects also played a significant role, with 80% of respondents considering logistical factors before making a CVAD recommendation (Table 2). The most frequently mentioned logistical factor was waiting time for placement (65%). The majority (57%) reported an average waiting time for PORT placement of >6 days, while for PICC placement 53% indicated a waiting time of <3 days (Figure 3). For 41% of PORT and 43% of PICC placements, the reported waiting time was 3-6 days.

**Table 2:** The impact of logistics, costs and complication risk on CVAD recommendation

	*n* (%)
Do health care professionals take logistics into account when recommending a certain CVAD?	91
Yes	73 (80)
No	18 (20)
What logistics do health care professionals consider?^b^	85
Waiting time for CVAD placement	59 (65)
Guidelines for needed form of sedation	13 (14)
Certain CVADs are unavailable at their institution	4 (4)
Other	9 (10)
Do health care professionals take costs into account when recommending a certain CVAD?^b^	90
Yes	5 (6)
No	44 (49)
I don’t know what the costs are	41 (46)
Under which circumstances are PORTs placed?^b^	79
Under local anesthesia	15 (19)
Under sedation	14 (18)
Under general anesthesia	20 (25)
All options are possible, the patient decides based on their preference	14 (18)
All options are possible, the oncology clinician decides based on their experience and preference	3 (4)
I don’t know	13 (17)
Other	0
Under which circumstances are PICCs placed?^b^	86
Under local anesthesia	80 (93)
Under sedation	0
Under general anesthesia	0
All options are possible, the patient decides based on their preference	2 (2)
All options are possible, the oncology clinician decides based on their experience and preference	0
I don’t know	4 (5)
Other	0
Do health care professionals explain the complication risk of the different CVAD options?^b^	85
Never	1 (1)
Rarely	9 (11)
Sometimes	10 (12)
Often	21 (25)
(Almost) always	44 (52)
For what reason(s) do health care professionals hesitate to explain complication risks?^b^	37
Lack of good overview	10 (27)
Lack of time	7 (19)
Don’t have all the current knowledge to explain this	8 (22)
Other^a^	12 (32)
According to the experience of health care professionals, how often do patients on average experience complications with PORTs?^b^	82
Never	1 (1)
Rarely	39 (48)
Sometimes	37 (45)
Often	2 (2)
(Almost) always	0
I don’t have experience with PORTs	3 (4)
According to the experience of health care professionals, how often do patients on average experience complications with PICCs?^b^	89
Never	0
Rarely	16 (18)
Sometimes	62 (70)
Often	10 (11)
(Almost) always	1 (1)
I don’t have experience with PICCs	0

CVAD, central venous access device; PICC, peripherally inserted central catheter; PORT, port-a-cath.

a Other reasons include explanation by surgeon/radiologist (n = 4), only one CVAD option available (*n* = 6), and deeming the information too extensive for patients and irrelevant for the decision-making process (*n* = 2).

b Not all numbers add up to 91 as some respondents did not answer all questions.

Another logistical factor considered by 14% of health care professionals was the requirement for anesthesia. PICC placement was predominantly carried out under local anesthesia (93%), with 2% of respondents indicating that patients could also opt for sedation or general anesthesia (Table 2). In contrast, PORT placement showed greater variability: 19% reported PORT placement under local anesthesia only, 18% under sedation, and 25% under general anesthesia. In 22% of cases, multiple anesthesia options were available. Notably, 17% and 5% of respondents, respectively, were unaware of the anesthesia requirements for PORT and PICC placement at their institutions.

Other logistical factors mentioned influencing CVAD choice included the availability of a double lumen for concurrent total parenteral nutrition initiation, stock availability, operating room capacity, accessibility of blood draws at external locations, and scheduling convenience between radiology and surgical departments.

Cost consideration played a minor role in decision making, with only 6% of respondents considering financial aspects in CVAD selection. Nearly half (49%) reported not taking costs into account, while 46% reported being unaware of the associated costs.

### Complication risk and CVAD selection

Risk of infection and thrombosis were key considerations for 45% and 35% of respondents, respectively (Figure 2). Among those who preferred PORTs, 66% and 43% considered infection risk and thrombosis risk, respectively, making these two of the top five determinants in their decision making, next to patient comfort (60%), patient preference (51%), and treatment duration (46%). For those favoring PICCs, complication risks were not among the top five decision-making factors, but they did consider patient preference (64%), waiting time for placement (62%), treatment duration (55%), ease of placement (51%), and patient comfort (47%) as most important influence on their CVAD choice. Thus, while several considerations were reported by both groups, safety-related factors primarily distinguished clinicians preferring PORTs, whereas logistical and procedural considerations were more prominent among those favoring PICCs.

According to personal experiences, most respondents described complications as ‘rare’ with PORTs (48%), while PICC complications occurred ‘sometimes’ (70%) or ‘often’ (11%). Complication risks were (almost) always discussed by 52%, while 25% did so often, 12% sometimes, and 12% rarely to never. The most common reasons for not discussing complications included a lack of good overview of common complications (27%), insufficient knowledge (22%), and time constraints (19%). Additional reasons included explanation by surgeons/radiologists, irrelevance to the decision-making process, or the availability of only one CVAD option.

### Shared decision making

**Figure 4:**
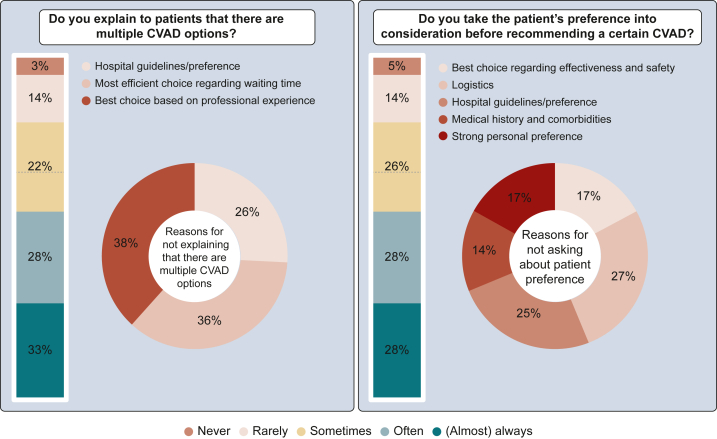
**Shared decision making in CVAD selection.** CVAD, central venous access device.

Respondents were divided regarding whether they routinely informed patients about there being multiple CVAD options. While 33% stated they almost always explained the available options, 28% did so often, 22% sometimes, and 17% rarely or never (Figure 4). The main reasons for not explaining that there are multiple CVAD options were personal experience with complication rates (38%), prioritizing efficiency based on waiting time (36%), and hospital guidelines restricting available options (26%).

Despite patient preference being identified as the most influential factor in CVAD selection, only 28% of respondents (almost) always considered patient preference when making a CVAD recommendation. Another 28% often took it into account, 26% sometimes, and 19% rarely or never considered patient preference. The primary reasons for not engaging in SDM included logistical constraints (27%), hospital guidelines (25%), strong preference for a particular CVAD based on effectiveness/safety (17%), due to an unknown reason (17%), and patient comorbidities (14%).

According to respondents, the most important factors influencing patient preference included comfort during use (63%), invasiveness of placement (54%), waiting time for placement (41%), complication risk (39%), and the need for sedation (24%).

## Discussion

This study, including the perspectives of 91 oncology professionals, revealed significant variation in CVAD availability, decision-making autonomy, CVAD selection, and incorporation of evidence and patient preferences. These findings highlight opportunities to improve both the quality of care and quality of life of patients with pancreatic cancer by better understanding real-world decision-making contexts.

Although most respondents had access to both PORTs and PICCs, 12% could only offer one type of CVAD. This aligns with previous research, with a British randomized trial experiencing difficulties in randomizing between PORTs, PICCs, and HCs across various centers because of availability issues.^12^ Also, hospital-wide guidelines or preferences dictated CVAD selection for nearly half of respondents, further limiting clinician and patient autonomy. Yet, these guidelines varied greatly as both PORTs (42%) and PICCs (51%) were named hospital preference. Notably, respondents rarely disagreed with their institutional guideline, suggesting that institutional policies shape practice patterns despite a lack of CVAD-specific recommendations in national and international guidelines.^8^^,^^18^^,^^20^

The absence of clear guideline recommendations seems to partly explain the wide variability in individual practice. Treatment duration was another factor of influence on CVAD choice for nearly half of respondents, with PICCs being preferred for short-term and PORTs for longer term use—despite the lack of clear cut-off points. Reported thresholds for PICC dwell time range from weeks to 12 months. A study on PICC-associated bloodstream infections identified 25 days as the optimal catheter duration^21^ and data from the CAVA three-arm randomized controlled trial also claimed that PORTs are favorable for palliative chemotherapy administration with an expected duration exceeding 3 months due to lower complication rates at similar costs.^11^ These time spans are both shorter than typical chemotherapy regimens for pancreatic cancer. However, it is important to recognize that in pancreatic cancer, particularly in the metastatic setting, median treatment duration and overall survival are often limited, and both treatment tolerance and efficacy are uncertain. These disease-specific characteristics may partly explain why clinicians still consider PICCs a pragmatic option in daily practice.

Previous literature also consistently supports PORTs as the safer and more durable option when compared with PICCs. Meta-analyses based on randomized controlled trials all show that PICCs are associated with higher overall complication rates, including thrombosis, device failure, unplanned removal, and local reactions, while PORTs mainly carry risks of local site infections (without septicemia and pain).^11^, ^12^, ^13^^,^^16^^,^^22^, ^23^, ^24^, ^25^, ^26^ Evidence concludes that PORTs are the safer and more comfortable CVAD option for cancer patients undergoing systemic therapy. Also in practice, oncology specialists appear to acknowledge complication risk among one of the key factors influencing CVAD choice. However, interestingly, those who preferred PORTs more often listed infection and thrombosis risks among their top five decision-making factors compared with PICC prescribers. Given that literature generally suggests higher complication rates associated with PICCs, it is notable that clinicians who prefer PICCs did not seem to prioritize these in their selection process.^11^, ^12^, ^13^ This discrepancy could be due to different risk perceptions or institutional protocols. Moreover, one in four clinicians lacked a clear overview of complication risks, and one in five did not feel equipped to properly educate patients. Others noted that explaining complications was the responsibility of the surgeon or radiologist, implying that the choice of CVAD had already been made before complications were discussed. Some clinicians reported omitting complication discussions because they deemed the information too complex for patients. However, previous research has shown that risks such as thrombosis and infection significantly influence patients on CVAD choice.^11^, ^12^, ^13^^,^^27^ Given the hypercoagulable state associated with pancreatic cancer, underestimating thrombotic risk may expose patients to avoidable harm.^28^

Procedural feasibility and anesthesia requirements further seemed of influence on decisions, mostly among PICC prescribers. PICCs were perceived as faster and less invasive, whereas PORTs often required longer scheduling times and anesthesia. Our survey revealed striking variability in anesthesia: one in five PORTs were routinely placed under local anesthesia, one in five under conscious sedation, and one in four under general anesthesia. Only 18% of respondents stated that patients could choose their preferred form of anesthesia for PORT placement, and almost one in five clinicians were unaware of which type of sedation was used at their institution. This raises concerns about how clinicians can adequately inform patients about CVAD options if they lack knowledge of procedural details. Furthermore, the possible relatively frequent use of general anesthesia in the Netherlands contrasts with international practice, where PORTs are rarely placed under general anesthesia.^29^, ^30^, ^31^ Requiring general anesthesia weakens the choice for PORTs and highlights the need to improve the logistics of PORT placement. One promising strategy might be the implementation of nurse-led vascular access teams, which could reduce waiting times and procedural burden while expanding access to CVADs.

Beyond procedural concerns, logistical constraints in general, particularly waiting times, also emerged as key drivers of CVAD selection. For more than half of respondents, PICCs were typically placed within 3 days, while PORT placement exceeded 6 days for more than half. While our data does not allow assessment of whether this practice variation represents appropriate clinical flexibility or unwarranted inconsistency, a previous study similarly reported that lack of available slots for PORT insertion often drove PICC selection.^12^ Yet, if the medical condition allows, the decision for a ‘faster’ CVAD should be shifted from the health care professional to the patient, ensuring full transparency regarding risks and benefits of alternative options.

Despite the evidence of higher complication risks and shorter safe dwell times, our study shows nearly half of oncology clinicians use PICCs in daily practice for the administration of systemic therapy in pancreatic cancer patients. This discrepancy may reflect a combination of contextual factors and disease-specific characteristics. In metastatic pancreatic cancer, where prognosis is poor and the urgency to initiate treatment is high, clinicians may be more inclined to consider a PICC when this allows faster initiation. Similarly, the frequent uncertainty about whether patients will tolerate or benefit from intensive chemotherapy regimens also contributes; in such cases clinicians may be reluctant to commit to a PORT upfront in situations where early treatment discontinuation is anticipated. In addition, institutional experience with PICCs, particularly their common use for short-term indications such as antibiotics, may inappropriately perpetuate their use in oncology for longer term indications such as systemic therapy administration. Finally, the upcoming routine use of prophylactic-dose anticoagulation in pancreatic cancer may reduce clinicians’ concern about thrombotic events.

A last possible explanation for PICC use might be the incorporation of SDM, as patient preference was also mentioned as one of the most influential factors in CVAD selection by more than half of respondents. However, in practice it appears that patient preference was often outweighed by logistical factors: only one in three clinicians (almost) always informed patients about multiple CVAD options, and only one in four consistently incorporated patient preference into their recommendations. Barriers to fully implement SDM included institutional guidelines or preferences, procedural waiting times, and clinical preference based on safety concerns, comorbidities, and personal experience according to respondents. These findings suggest that patient preference might be inconsistently applied in practice. From the patient perspective, studies show that awareness of multiple CVAD options is associated with greater satisfaction and acceptance, regardless of the device chosen.^13^^,^^24^ While studies show broadly similar quality of life with both devices, PORTs are often preferred by patients in terms of daily functioning, hobbies, self-consciousness, and socializing.^12^^,^^22^^,^^23^^,^^32^ Still, in pancreatic cancer, safety and treatment feasibility must remain the primary drivers of CVAD selection, with patient preference and comfort serving as a complementary factor.^12^^,^^33^

While this study benefits from a diverse and nationally representative clinician sample, limitations include, firstly, potential recall bias and social desirability bias as the study is based on self-reported data. Secondly, the questionnaire was designed as an exploratory expert survey and did not undergo formal psychometric validation, which may affect measurement precision. Also, the exploratory design does not allow causal inference regarding the drivers of CVAD selection. Thirdly, the study primarily included respondents from the Netherlands to reflect national practice, limiting international generalizability. Given the centralized organization of pancreatic cancer care in the Netherlands, the number of respondents likely captures a substantial proportion of clinicians involved in pancreatic cancer treatment nationwide. However, due to open dissemination via professional societies, we were unable to calculate a precise response rate and thus cannot prevent potential nonresponse bias. Fourthly, the study did not focus on patient-reported outcomes, which could provide further context regarding the impact of CVAD selection on patient experience.

In conclusion, while PORTs remain the evidence-based choice for long-term safety, durability, and reliability, particularly relevant in the thrombogenic pancreatic cancer population, our findings reflect that daily practice is still often shaped by both logistical constraints and disease-specific factors such as poor prognosis, urgent treatment initiation, and uncertainty regarding treatment tolerance. PICCs therefore continue to be widely used, not out of disregard for evidence, but due to these real-world considerations and local expertise. Although this may expose patients to avoidable risks, it underscores the importance of improving patient counseling, reducing logistical barriers, and expanding institutional capacity for CVAD placement. Strengthening anesthesia protocols, fostering clinician awareness, and developing clearer guideline recommendations may help bridge the gap between evidence and practice. Ultimately, aligning clinical decisions with both evidence and real-world constraints can support safer, more individualized care and improve quality of life for patients undergoing chemotherapy for pancreatic cancer.
