# Supplementary material for: Preference versus protocol: oncology clinicians’ perspectives on central venous access for administration of chemotherapy in pancreatic cancer
Source: ESMO Gastrointest Oncol. 2026 Mar 2;11:100311. doi: 10.1016/j.esmogo.2026.100311 (PMC12969377; doi:10.1016/j.esmogo.2026.100311)
Supplement: Supplementary Material [file mmc1.docx]

**SUPPLEMENTARY MATERIAL**

**Supplementary tables**

**Supplementary table 1: Survey on central venous access devices (CVADs) in patients with pancreatic cancer**

| Dear colleague, We kindly invite you to participate in this survey aimed at gaining more insight into current clinical practice regarding central venous access devices (CVADs) in patients with pancreatic cancer. CVADs refer to central venous access ports used for administering 5-FU–based therapy (e.g., port-a-cath, PICC line, etc.). The questionnaire will take no more than 5 minutes of your time, and all responses will be processed anonymously. Thank you in advance for your valuable contribution! | | | | | | | | |  |
| --- | --- | --- | --- | --- | --- | --- | --- | --- | --- |
| 1 | What is your profession? | | | | | | | |  |
|  | Medical oncologist  Medical oncologist in training  Nurse specialist  Physician assistant  Other, namely: | | | | | | | |  |
| 2 | What is your current working environment? | | | | | | | |  |
|  | Academic hospital  Non-academic tertiary referral center  Non-academic hospital  Other, namely: | | | | | | | |  |
| 3 | In what country do you practice? | | | | | | | |  |
|  | The Netherlands  Belgium  Other, namely: | | | | | | | |  |
| 4 | What is your experience with working in the field of pancreatic cancer, given in years? | | | | | | | |  |
|  | < 5 years  5-10 years  > 10 years | | | | | | | |  |
| 5 | How many new patients with pancreatic cancer do you treat on average on an annual basis? | | | | | | | |  |
|  | <10  10-30  >30 | | | | | | | |  |
| 6 | Do you play a role in the recommendation/placement of CVADs in your patients? | | | | | | | |  |
|  | Yes  No (end of survey) | | | | | | | |  |
| 7 | Which CVADs are available for the administration of 5-Fluorouracil (5-FU) in patients with pancreatic adenocarcinoma in your hospital? | | | | | | | |  |
|  | Port-a-cath (port or PAC)   Peripherally Inserted Central Catheter (PICC)  Hickman Catheter (HC)  Other, namely: | | | | | | | |  |
| 8 | In what percentage do you recommend each CVAD to your patients for the administration of 5-FU in patients with pancreatic adenocarcinoma? | | | | | | | |  |
|  |  | | Never | < 25% | 25-50% | 50-75% | > 75% | Always |  |
|  | Port-a-Cath | |  |  |  |  |  |  |  |
|  | PICC line | |  |  |  |  |  |  |  |
|  | Hickman catheter | |  |  |  |  |  |  |  |
|  | Other | |  |  |  |  |  |  |  |
| 9 | | | Do you follow hospital guidelines/protocols for the choice of a CVAD? | | | | | | |
|  |  |  | Yes, I follow hospital guidelines/protocol and can only use the CVAD the hospital recommends  🡪 If yes: Would your approach or recommendation regarding CVAD selection be different if you did not have to take hospital guidelines or protocols into account?  No  Yes:  Yes, there is a hospital-wide preference, and I largely follow it  No, I am allowed to follow my own preference | | | | | | |
| 10 | | | What are the **five** most important factors you consider when advising on or selecting a type of CVAD? | | | | | | |
|  |  |  | Risk of venous thrombosis  Risk of infection  Risk of device malfunctioning  Durability of the device  Patient comorbidities/medical history  Need for sedation/perioperative risk  Patient preference   Patient comfort   Possibility of taking blood samples  Hospital guidelines/preference  Waiting time for placement  Costs  My own experience  Ease of placement  Frequency of CVAD care  Treatment duration | | | | | | |
| 11 | | | In your experience, how often in the past year have patients experienced complications with a port-a-cath (port or PAC)? | | | | | | |
|  |  |  | (Almost) always  Often  Sometimes  Rarely  Never  I haven’t prescribed PACs | | | | | | |
| 12 | | | What complications have you seen most often in patients with PACs? | | | | | | |
|  |  |  | Venous thrombosis  Infection  Device malfunction  Insertion complication   Occlusion  Patient dissatisfaction/discomfort  Other, namely:        I don’t know | | | | | | |
| 13 | | | In your experience, how often in the past year have patients experienced complications with a peripherally inserted central catheter (PICC)? | | | | | | |
|  |  |  | (Almost) always  Often  Sometimes  Rarely  Never  I haven’t prescribed PICCs | | | | | | |
| 14 | | | What complications have you seen most often in patients with PICCs? | | | | | | |
|  |  |  | Venous thrombosis  Infection  Device malfunction  Insertion complication   Occlusion  Patient dissatisfaction/discomfort  Other, namely:        I don’t know | | | | | | |
| 15 | | | I take hospital logistics into account before recommending a certain CVAD (eg. Waiting list, ...) | | | | | | |
|  |  |  | Yes  No, I advise every patient in the same manner  🡪 If yes: What hospital logistics have an influence on CVAD choice:  Waiting time  Guideline regarding a specific form of required sedation   Recommending a specific CVAD is not possible/uncommon in my center  Other, namely: | | | | | | |
| 16 | | | What is the average waiting time for PAC placement in your hospital? | | | | | | |
|  |  |  | < 3 days  3-6 days  > 6 days | | | | | | |
| 17 | | | What is the average waiting time for PICC placement in your hospital? | | | | | | |
|  |  |  | < 3 days  3-6 days  > 6 days | | | | | | |
| 18 | | | Do you consider CVAD costs before recommending a certain CVAD | | | | | | |
|  |  |  | Yes 🡪 If yes: What CVAD do you find most cost-effective?        No  No, I am not aware of the cost implications | | | | | | |
| 19 | | | In your hospital, under which circumstances is a port-a-cath (PAC) placed? | | | | | | |
|  |  |  | Under local anesthesia  Under sedation (propofol/’roesje’)  Under general anesthesia (narcose)  All options are possible, the patient decides based on their preference  All options are possible, I decide based on my experience and preference  I don’t know  Other, namely: | | | | | | |
| 20 | | | In your hospital, under which circumstances is a PICC placed? | | | | | | |
|  |  |  | Under local anesthesia  Under sedation (propofol/’roesje’)  Under general anesthesia (narcose)  All options are possible, the patient decides based on their preference  All options are possible, I decide based on my experience and preference  I don’t know  Other, namely: | | | | | | |
| 21 | | | Do you explain to the patient that there are multiple options in central venous access devices? | | | | | | |
|  |  |  | (Almost) always  Often  Sometimes  Rarely  Never | | | | | | |
| 22 | | | For what reason(s) do you not inform all patients that there are multiple CVAD options? | | | | | | |
|  |  |  | According to hospital protocol, there is only one option  I choose the most efficient option in terms of waiting time   I base my choice on my own experience with complications  Other or explanation: | | | | | | |
| 23 | | | Do you explain the complication risks of the multiple CVAD options? | | | | | | |
|  |  |  | (Almost) always  Often  Sometimes  Rarely  Never | | | | | | |
| 24 | | | For what reason(s) do you not explain complication risks of multiple CVAD options to all patients? | | | | | | |
|  |  |  | Lack of proper overview  Lack of time  I do not have all the current knowledge to explain this Other, namely: | | | | | | |
| 25 | | | Do you take patient preference into account before recommending a CVAD? | | | | | | |
|  |  |  | (Almost) always  Often  Sometimes  Rarely  Never | | | | | | |

| 26 | What reason(s) prevent you from taking patient preference into account? |
| --- | --- |
|  | Due to logistical reasons  Strong personal preference (of the healthcare provider)  Due to medical history/comorbidities  In my opinion, one specific CVAD is preferred due to effectiveness/safety/...  Other, namely: |
| 27 | In your experience, what are the most important factors patients take into consideration when making a CVAD choice? |
|  | Invasiveness of the procedure  (Dis)comfort during use  Risk of complications   Waiting time until placement  Need for sedation  Other, namely: |

**Supplementary figures**


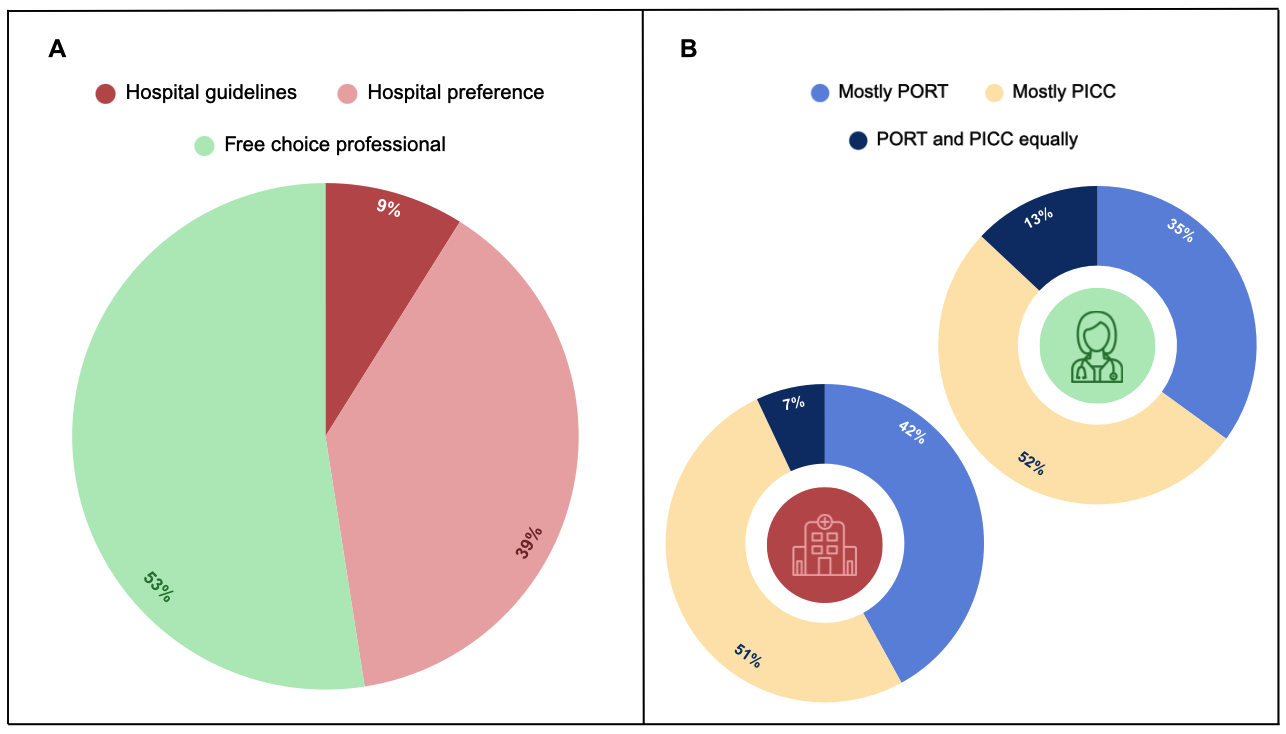


**Supplementary figure 1: decision-making autonomy and CVAD preference**
S1-A: Possibility to recommend CVAD of preference or need to follow hospital guidelines or a hospital-wide preference
S1-B: What CVAD is most often selected by healthcare professionals who have to follow hospital guidelines/preference versus who have free CVAD choice

*Abbreviations: CVAD = central venous access device, PORT = port-a-cath, PICC = peripherally Inserted Central Catheters*
