# Supplementary figures and images for: Preference versus protocol: oncology clinicians’ perspectives on central venous access for administration of chemotherapy in pancreatic cancer
Source: ESMO Gastrointest Oncol. 2026 Mar 2;11:100311. doi: 10.1016/j.esmogo.2026.100311 (PMC12969377; doi:10.1016/j.esmogo.2026.100311)

**A**

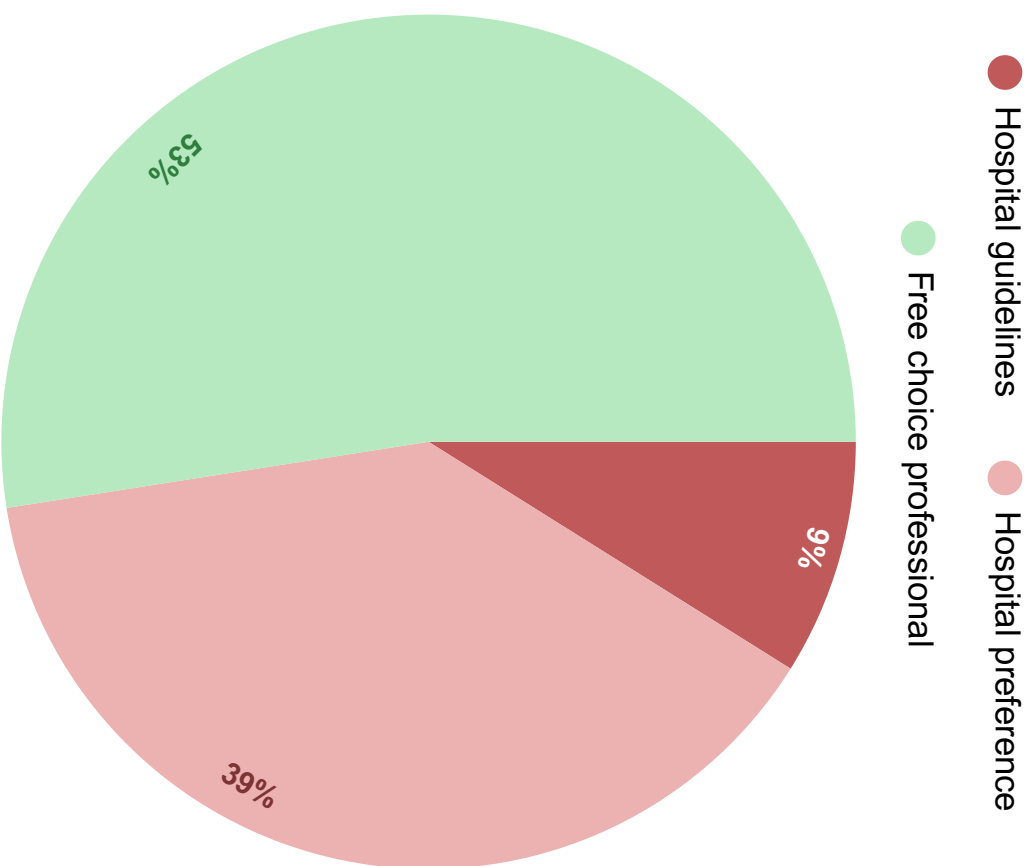

**B**

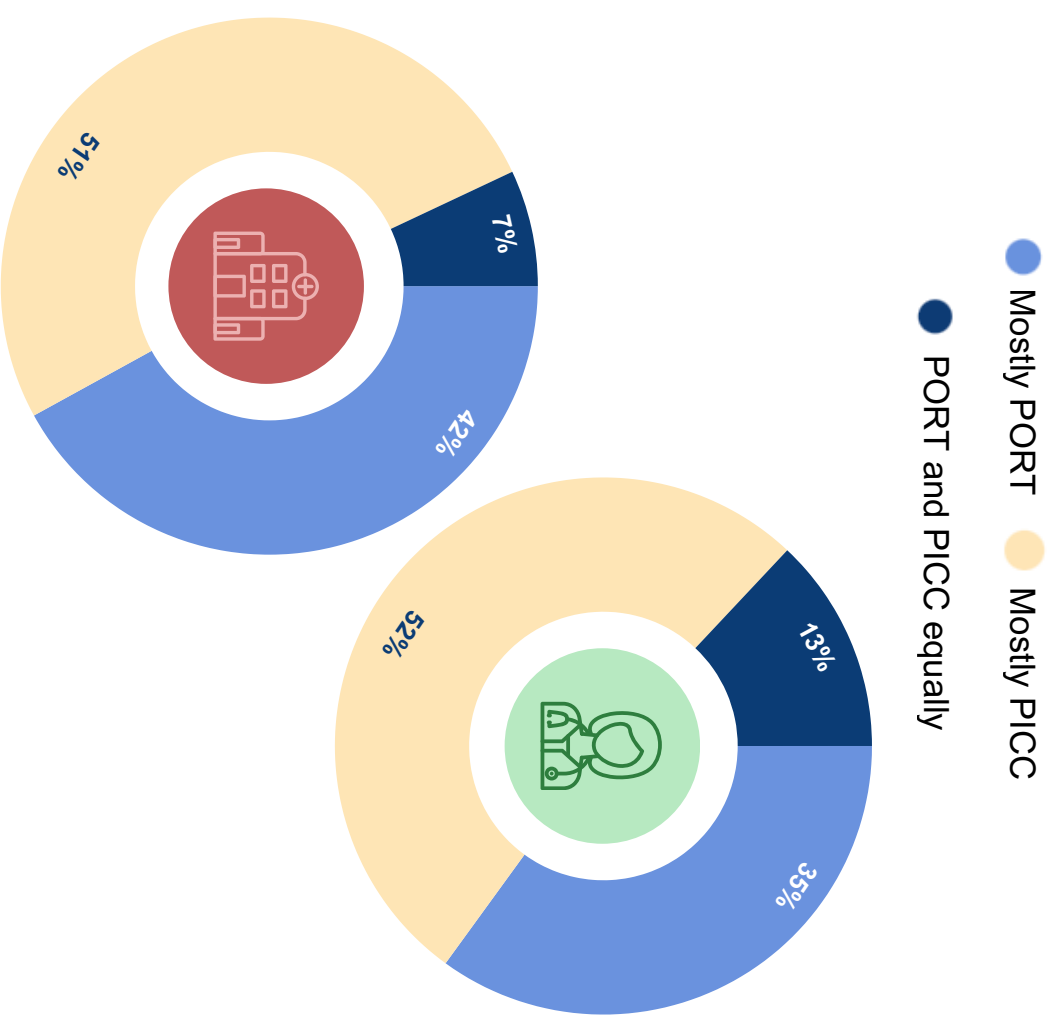

Supplement: Supplementary Figure 1 [file mmc2.pdf]
